# Depression pathogenesis and treatment: what can we learn from blood mRNA expression?

**DOI:** 10.1186/1741-7015-11-28

**Published:** 2013-02-05

**Authors:** Nilay Hepgul, Annamaria Cattaneo, Patricia A Zunszain, Carmine M Pariante

**Affiliations:** 1 Sections of Perinatal Psychiatry & Stress, Psychiatry and Immunology, Department of Psychological Medicine, Institute of Psychiatry, King's College London, 125 Coldharbour Lane, London, SE5 9NU, UK

**Keywords:** depression, gene expression, glucocorticoid receptor, inflammation, microarray, mRNA, neuroplasticity, peripheral blood, transcriptomics

## Abstract

Alterations in several biological systems, including the neuroendocrine and immune systems, have been consistently demonstrated in patients with major depressive disorder. These alterations have been predominantly studied using easily accessible systems such as blood and saliva. In recent years there has been an increasing body of evidence supporting the use of peripheral blood gene expression to investigate the pathogenesis of depression, and to identify relevant biomarkers. In this paper we review the current literature on gene expression alterations in depression, focusing in particular on three important and interlinked biological domains: inflammation, glucocorticoid receptor functionality and neuroplasticity. We also briefly review the few existing transcriptomics studies. Our review summarizes data showing that patients with major depressive disorder exhibit an altered pattern of expression in several genes belonging to these three biological domains when compared with healthy controls. In particular, we show evidence for a pattern of 'state-related' gene expression changes that are normalized either by remission or by antidepressant treatment. Taken together, these findings highlight the use of peripheral blood gene expression as a clinically relevant biomarker approach.

## Introduction

Major depressive disorder (MDD) is a complex disorder characterized by the interaction between biological, genetic and environmental factors, and by a pathogenesis involving alterations in several biological systems. A large amount of research has been focused on understanding the underlying mechanisms of MDD, and there is already a wealth of evidence demonstrating changes not only in the central nervous system (CNS) but also in the periphery. For example, blood and saliva are useful and accessible systems that, via relatively low-invasive procedures, can be used to analyze several biomarkers, such as proteins or metabolites, using quantitative techniques. Using this approach, hormonal and immunological abnormalities, such as elevated levels of pro-inflammatory cytokines [1,2], alterations of the hypothalamic-pituitary-adrenal (HPA) axis [3], changes in neuroplasticity [4], and changes in oxidative and nitrosative stress pathways [5], have all been documented in patients with MDD, and are indicative of the 'neuroprogressive' nature of MDD [6].

An emerging and useful method to investigate the pathogenesis of this disorder is the use of peripheral blood to measure the expression levels of genes. This is a useful approach in biomarker identification, with opportunities for both hypothesis-driven biomarker search and for hypothesis-free transcriptomics-based discovery [7]. 'Blood gene expression' usually refers to intracellular RNA from blood, and it is technically associated, in most cases, with two approaches: the use of tubes for blood collection that stabilize mRNA from all cells in the blood; and the extraction of mRNA from separate distinct blood cell populations. What is really of importance for researchers is whether blood mRNA can be used as a proxy for mRNA expression in other tissues that are more relevant to the pathogenic processes of interest - in psychiatry and neuroscience, the brain. In this regard, peripheral blood gene expression is very promising, as several studies have shown that blood cells share more than 80% of the transcriptome with other body tissues, including the brain [8]. For example, Sullivan and colleagues compared the transcriptional profiling of 79 human tissues, including that of whole blood and of several brain areas. They showed that whole blood shares significant gene expression similarities with multiple brain tissues, in particular for genes encoding for neurotransmitter receptors and transporters, stress mediators, cytokines, hormones, and growth factors, all of which are relevant to MDD [9]. As such, investigating peripheral blood gene expression appears to be a useful tool for assessing and understanding MDD.

It is important to mention that biomarker researchers have previously used the term leukocyte gene expression to refer to blood gene expression, implying that the mRNA isolated from blood comes predominantly from leukocytes (white blood cells) - that is, the cells of the immune system. This assumption has been largely based on the notion that erythrocytes, or red blood cells, even if much more abundant than leukocytes (by a factor of approximately 1,000), do not have a nucleus and as such should not have mRNA synthesis. However, most recent research has suggested that, in fact, whole blood mRNA comes predominantly from erythrocytes [7].

As mentioned above, there is clear evidence that biological systems such as the HPA axis and the inflammatory response are altered and can contribute to the pathogenesis of depression [10,11]. The dysfunction of these systems is largely thought to be a result of the activation of stress-related mechanisms, as MDD is often preceded by acute or chronic stressful experiences [12]. We and others have proposed an explanatory model centered on the glucocorticoid receptor (GR), one of the most important receptors and transcription factors governing the stress response [3]. Stress can induce glucocorticoid resistance, that is, a reduction of GR function, which in turn leads to both HPA axis hyperactivity and increased inflammation. As communication occurs between the CNS and the endocrine and immune systems, an activation of one can affect the processes of another, and vice versa [13]. Based on this, we review the current literature on blood gene expression alterations in MDD. Additionally, we look at bipolar disorder (BPD) studies that consider euthymic or depressed states, but excluding those that look at manic states. We focus in particular on three important and interlinked domains: inflammation, GR functionality and neuroplasticity. We also briefly review the few existing transcriptomics studies.

## Alterations in the expression of genes involved in inflammation

The inflammatory theory of MDD emphasizes the role of psychoneuroimmunological dysfunctions where there is an activation of the immune system [14-17]. Moreover, MDD is very common in the medically ill, particularly in conditions with an inflammatory component, such as cardiovascular disease and rheumatoid arthritis, as well as in autoimmune and neurodegenerative disorders [18,19]. Indeed, patients with MDD have been consistently shown to have altered levels of pro- and anti- inflammatory cytokines in circulation [1,2,20], and postmortem studies have also described gene expression alterations in a variety of these cytokines [21]. However, studies on postmortem brains have several limitations that can affect the results, including the brain region analyzed, the cause of death and the effect of the antidepressant treatments on gene expression. As such, researchers are using peripheral tissues, such as leukocytes, that have several advantages, which we have already mentioned [9]. A few studies have assessed the mRNA levels of genes involved in inflammation in the peripheral blood of patients with MDD (see Table 1). For example, Tsao and colleagues found that the expression of IL-1β, IL-6, TNF-α and IFN-γ genes was significantly higher in the peripheral blood mononuclear cells of patients with MDD compared with healthy controls [22]. Moreover, in a sub-sample of patients, they demonstrated that IFN-γ expression was reduced, though not normalized, after 3 months of fluoxetine treatment, suggesting that antidepressants may have anti-inflammatory properties. Similarly, we have recently shown altered gene expression levels in a number of cytokines in the whole blood of patients with MDD when compared with controls [23]. We have found higher mRNA levels of IL-1β, IL-6, TNF-α and macrophage inhibiting factor (MIF) as well as lower levels of IL-4. Moreover, we have also found that IL-β, TNF-α and MIF mRNA levels are predictors of antidepressant treatment response, as all three show higher baseline expression in non-responders. Finally, we also demonstrated that IL-6 expression is reduced after 8 weeks of antidepressant treatment (escitalopram or nortriptyline) in responders only, suggesting a unique ability of these biomarkers to both predict and track the therapeutic antidepressant response.

**Table 1:** Studies examining alterations in the expression of genes involved in inflammation

Citation	Type of study	Sample	Gene	Clinical assessments	Main findings
Tsao *et al*. 2006 [22]	Case-control and follow-up	20 MDD 22 Controls	*5-HTT* *IL-1β* *IL-6* *TNF-α* *IFN-α*	Diagnosis based on DSM-IV criteria HDRS	Higher expression of *5-HTT, IL-1β, IL-6, TNF-α *and *IFN-α *in patients with MDD compared with controls. Significant decreased in expression of *5-HTT *and *IFN-α *after 3 months of fluoxetine treatment.
Cattaneo *et al*. 2012 [23]	Case-control and follow-up	74 MDD 34 Controls	*IL-1α* *IL-1β* *IL-4* *IL-6* *IL-7* *IL-8* *IL-10* *MIF* *TNF-α*	Diagnosis based on DSM-IV or ICD-10 criteria SCAN	Higher expression of *IL-1β, IL-6, MIF *and *TNF-α *and lower expression of *IL-4 *in patients with MDD compared with controls. *IL-β, TNF-α *and *MIF *expression predicted antidepressant response. Significant decrease in *IL-6 *expression after 8 weeks of antidepressant treatment (escitalopram or nortriptyline) in responders only.
Suzuki *et al*. 2010 [25]	Case-control	43 MDD 43 Controls	*ApoER2* *VLDLR*	Diagnosis based on DSM-IV criteria HAM-D BPRS	Lower expression of *ApoER2 *in patients with MDD compared with controls. No significant difference in expression of *VLDLR*.
Galecki *et al*. 2012 [29]	Case-control	181 rDD 149 Controls	*COX-2* *MPO* *NOS2A* *PLA2G2A*	Diagnosis based on DSM-IV criteria or ICD-10 criteria CIDI	Higher expression of *COX-2, MPO*, *NOS2A *and *PLA2G2A *in patients with rDD compared with controls.
Weigelt *et al*. 2011 [33]	Case-control	24 MDD 22 BPD 45 Controls	*TREM-1* *DAP12* *PU.1* *ATF3* *EGR3* *MXD1* *MAFF* *NAB2*	Diagnosis based on DSM-IV criteria SCID-I	Higher expression of *TREM-1 *and *NAB2 *in patients with BPD only. Higher expression of *PU.1 *in patients with MDD and a trend for higher expression of *TREM-1*. Higher expression of *ATF3, EGR3, MAFF *and *MXD1 *in patients with both MDD and BPD. No significant difference in expression of *DAP12 *in either patient group.

BDI: Beck Depression Inventory; BPD: bipolar disorder; BPRS: Brief Psychiatric Rating Scale; CIDI: Composite International Diagnostic Interview; DSM-IV: Diagnostic and Statistical Manual, Fourth Edition; HAM-D: Hamilton Rating Scale for Depression; HDRS: Hamilton Depression Rating Scale; ICD-10: International Classification of Diseases - 10^th ^revision; MDD: major depressive disorder; rDD: recurrent depressive disorder; SCAN: Schedules for Clinical Assessment in Neuropsychiatry, SCID-I: Structured Clinical Interview for DSM-IV Axis I Disorder..

The activation of the immune system observed in patients with MDD is of course not limited to changes in cytokines production. For example, apolipoprotein E (ApoE) is a protein produced by macrophages known to act as an immunomodulator. ApoE is thought to interact with many immunological processes including suppression of T cell proliferation, macrophage function regulation and activation of natural killer T cells [24]. One study investigated the expression levels of the ApoE receptor, ApoER2, in lymphocytes of patients with MDD and healthy controls [25], demonstrating that patients with MDD had a significantly lower expression of ApoER2 compared with controls. These receptors bind to reelin, an extracellular matrix glycoprotein that plays crucial roles in brain development as well as in synaptic plasticity in the adult brain [26]. Interestingly, blood levels of an isoform of reelin have also been shown to be reduced in patients with MDD [27].

There is also gene expression evidence for the involvement of key inflammatory enzymes, including cyclooxygenase-2 (COX-2), myeloperoxidase (MPO) and inducible nitric oxide synthase, in the development of MDD [28-30]. These enzymes have been shown to be expressed not only in immune cells but also in the CNS. Moreover, increased oxidative and nitrosative stress as sequels of inflammation have been previously demonstrated postmortem as well as in animal studies [5,31]. Indeed, a gene expression study showed higher mRNA levels of COX-2, MPO, inducible nitric oxide synthase-2A and phospholipase A2 (PLA2G2A) in patients with MDD compared with healthy controls [32]. Furthermore, an increase in either reductive or oxidative stress is thought to be involved in the alteration of the expression of several neurotrophic factors, and will be reviewed below (see neuroplasticity).

Finally, a recent study in patients with MDD and euthymic BPD disorder investigated three key genes involved in inflammatory processes: triggering receptor expressed on myeloid cells 1 (TREM-1), DNAX-activation protein of 12 kDa (DAP12), and purine-rich Box-1 (PU.1) [33]. In this study, peripheral blood mononuclear cells were isolated from whole blood and gene expression carried out using purified monocytes. The results showed a significantly higher expression of PU.1 in patients with MDD and a significantly higher expression of TREM-1 in patients with BPD, with a trend for higher expression in patients with MDD compared with healthy controls, supporting, again, the role of inflammation in these disorders.

## Alterations in the expression of genes involved in glucocorticoid receptor functionality

Alterations of the HPA axis, including impairments in glucocorticoid-mediated negative feedback, is a well-established and consistent finding in MDD [34]. The GR is involved in this negative feedback and several studies have assessed GR expression and functionality in patients with MDD. These studies have primarily been conducted in peripheral cell types including immune cells (mononuclear and polymorphonuclear leukocytes) and fibroblasts (gingival and skin) [34]. Four studies have analyzed mRNA expression of GR or of GR-related molecules in peripheral blood (see Table 2). Katz *et al. *[35] investigated gene expression of chaperones and co-chaperones of the GR, such as FK506 binding protein (FKBP)-4 and FKBP-5, which influence GR function, and of GR target genes during pregnancy in individuals with a history of depression. They found an upregulation of eight genes during pregnancy in all patients; however, the expression of BAG family molecular chaperone regulator 1 (BAG1), FKBP-5, peptidylprolyl isomerase D (PPID) and nuclear receptor coactivator 1 (NCOA1) was reduced in mothers who were in a current depressive state. This suggests that maternal depression diminishes the pregnancy-related upregulation of these particular GR-related genes [35]. A part of these findings was replicated in our recent study, where we also assessed the expression of FKBP-4, FKBP-5 and GR in patients with MDD and controls [23]. We found higher mRNA levels of FKBP-5 and lower levels of GR in patients with MDD compared with controls. Furthermore, we found that antidepressant treatment significantly reduces FKBP-5 levels after 8 weeks in patients who responded to treatment, and increases GR levels in all patients, suggesting that a successful antidepressant treatment requires a normalization of GR function.

**Table 2:** Studies examining alterations in the expression of genes involved in GR functionality

Citation	Type of study	Sample	Gene	Clinical assessments	Main findings
Katz *et al*. 2012 [35]	Repeated measures	106 pregnant women with history of depression	*BAG1* *CDC37L1* *FKBP4* *FKBP5* *NCOR1* *NCOA1* *NR3C1* *HSP70* *ST13* *STIP1* *HSP90* *P23* *PPP5C* *PPIA* *PPID*	Diagnosis based on DSM-IV criteria SCID-I BDI	Higher expression of *BAG1, FKBP5, NCOA1 *and PPID during pregnancy was diminished in patients in a current depressive state.
Cattaneo *et al*. 2012 [23]	Case-control and follow-up	74 MDD 34 Controls	*FKBP-4* *FKBP-5* *GR*	Diagnosis based on DSM-IV or ICD-10 criteria SCAN	Higher expression of *FKBP-5 *and lower expression of *GR *in Patients with MDD compared with controls. Significant decrease in *FKBP-5 *expression and increase in *GR *expression after 8 weeks of antidepressant treatment (escitalopram or nortriptyline) in responders only.
Matsubara *et al*. 2006 [36]	Case-control	56 MDD 48 BPD 31 Controls	*GRα* *GRβ*	Diagnosis based on DSM-IV criteria HDRS	Lower expression of *GRα *in patients with MDD in a current depressive state and in remission compared with controls. Lower expression of *GRα *in patients with BPD in a current depressive state and in remission compared with controls. No significant differences in the expression of GRβ.
Fujimoto *et al*. 2008 [37]	Case-control	60 MDD 46 BPD 28 Controls	*Glo1*	Diagnosis based on DSM-IV criteria HDRS	Lower expression of *Glo1 *in patients with MDD and BPD in a current depressive state compared with controls. No significant difference in expression of *Glo1 *in patients with MDD and BPD in a remissive state compared with controls. Expression of *Glo1 *correlated negatively with depression scores.

BDI: Beck Depression Inventory; BPD: bipolar disorder; DSM-IV: Diagnostic and Statistical Manual, Fourth Edition; HAM-D: Hamilton Rating Scale for Depression; HDRS: Hamilton Depression Rating Scale; ICD-10: International Classification of Diseases - 10^th ^revision; SCAN: Schedules for Clinical Assessment in Neuropsychiatry; SCID-I: Structured Clinical Interview for DSM-IV Axis I Disorder.

In a third study, Matsubara *et al. *[36] investigated two isoforms of GR in both patients with MDD and with BPD: GRα, which is able to directly exert glucocorticoid effects, and GRβ, which binds poorly to glucocorticoids and, by forming heterodimers with GRα, impairs ligand binding of this isoform and acts as a dominant negative regulator of GR function [36]. The authors found that GRα expression was lower in patients with MDD and with BPD, in both current depressive states and in remission, compared with healthy controls. This suggests that GRα mRNA reduction is not state-dependent but a trait-dependent finding in mood disorders. These findings may seem at odds with the aforementioned study showing that antidepressant treatment increases GR expression [23]; however, it is important to note that most of the patients with depression in the study by Matsubara *et al*., even those defined as 'currently depressed', were already on antidepressants at the time of the gene expression analysis. They found no significant differences in the expression of GRβ in either patient groups compared with controls. Lastly, a study conducted by the same group, again in both patients with MDD and with BPD, investigated glyoxalase-1 (Glo1) [37], an antioxidant enzyme involved in oxidative stress and also a GR target gene as it contains consensus sequences for GR response elements [38]. It has been suggested that a GR dysfunction may also have an effect on Glo1 expression and, indeed, the authors found a lower expression of Glo1 in patients with MDD and BPD in a current depressive state compared with controls. On the contrary, there was no significant difference in Glo1 expression in patients with MDD or BPD in remission when compared with controls. This supports the notion that a reduced GR function, and thus a reduced expression of GR target genes like Glo1, is involved in the pathogenesis of depression, and that antidepressant treatment is able to restore this dysfunction. These data are also consistent with our experimental work showing that antidepressant treatment increases GR function both *in vivo *[39-42] and *in vitro *[43,44].

## Alterations in the expression of genes involved in neuroplasticity

MDD may also involve an inability of neuronal systems, especially under stress conditions, to show adaptive plasticity, a mechanism known as neuronal plasticity [4] (see Table 3). Molecular correlations underlying the mechanisms of the stress response involve the regulation of several neurotrophic factors, one of them being brain-derived neurotrophic factor (BDNF). To this regard, several studies have demonstrated reduced serum and plasma BDNF levels in patients with MDD when compared with controls, and now a few studies have investigated BDNF at gene expression level. Pandey *et al. *[45] investigated BDNF gene expression in both adult and pediatric patients with MDD and found significantly lower mRNA expression as well as lower protein levels in both MDD groups compared with controls [45]. These findings are supported by another of our studies, where we have also shown significantly lower BDNF expression in the peripheral leukocytes of patients with MDD compared with controls [46]. Additionally, we have found a significant increase in BDNF expression after treatment with the antidepressant escitalopram as well as a parallel improvement in depressive symptoms. In a similar study, we investigated the expression of the neuropeptide VGF (non-acronymic) in the peripheral leukocytes of patients with MDD and controls. VGF is known to be involved in synaptic plasticity and to be induced by BDNF [47], and we have shown that VGF expression is significantly lower in patients with MDD compared with controls [48]. Interestingly, we also found that expression of VGF is increased after 12 weeks of escitalopram treatment in those patients whose depressive symptoms were ameliorated. We have recently replicated these data in the aforementioned larger study [23], where again we show that patients with MDD have lower mRNA levels of BDNF and VGF, and that antidepressant treatment (escitalopram or nortriptyline) increases both BDNF and VGF expression in treatment responders.

**Table 3:** Studies examining alterations in the expression of genes involved in neuroplasticity

Citation	Type of study	Sample	Gene	Clinical assessments	Main findings
Pandey *et al*. 2010 [45]	Case-control	25 adult MDD 25 adult controls 14 pediatric MDD 14 pediatric controls	*BDNF*	Diagnosis based on DSM-IV criteria HDRS CARS-M CDRS YMRS	Lower expression of *BDNF *in both adult and pediatric patients with MDD compared with controls. Lower protein levels of *BDNF *in both adult and pediatric patients with MDD compared with controls.
Cattaneo *et al*. 2010a [46]	Case-control and follow up	21 MDD 23 Controls	*BDNF*	Diagnosis based on DSM-IV or ICD-10 criteria MADRS	Lower expression of *BDNF *in patients with MDD compared with controls. Significant increase in expression of *BDNF *after escitalopram treatment as well as symptom improvement.
Cattaneo *et al*. 2010b [48]	Case-control and follow up	25 MDD 25 Controls	*VGF*	Diagnosis based on DSM-IV or ICD-10 criteria MADRS	Lower expression of *VGF *in patients with MDD compared with controls. Significant increase in expression of *VGF *after 12 weeks of escitalopram treatment in responders only.
Cattaneo *et al*. 2012 [23]	Case-control and follow up	74 MDD 34 Controls	*BDNF* *VGF*	Diagnosis based on DSM-IV or ICD-10 criteria SCAN	Lower expression of *BDNF *and *VGF *in patients with MDD compared with controls. Significant increase in *BDNF *and *VGF *expression after 8 weeks of antidepressant treatment (escitalopram or nortriptyline) in responders only.
Otsuki *et al*. 2008 [49]	Case-control	60 MDD 42 BPD 28 Controls	*BDNF* *NGF* *NT-3* *NT-4* *GDNF* *ARTN* *NRTN* *PSPN*	Diagnosis based on DSM-IV criteria HDRS	Lower expression of *GDNF, ARTN *and *NT-3 *in patients with MDD in a current depressive state compared with those in remission and controls. No significant difference in expression of *GDNF, ARTN *or *NT-3 *in patients with BPD. No significant difference in expression levels of *BDNF, NGF *or *NT-3 *among the three groups.
Su *et al*. 2009 [54]	Case-control	16 MDD 14 Controls	*p11*	Diagnosis based on DSM-IV criteria MINI HAM-D HARS	Higher expression of *p11 *in patients with MDD compared with controls.
Zhang *et al*. 2011 [55]	Case-control	38 BPD 14 Controls	*p11*	Diagnosis based on DSM-IV criteria MINI HDRS-17 YMRS	Higher expression of *p11 *in patients with BPD compared with controls.
Iga *et al*. 2007 [57]	Case-control	42 MDD 32 Controls	*VEGF*	Diagnosis based on DSM-IV criteria SIGH-D 17	Higher expression of *VEGF *in patients with MDD compared to controls.
Dome *et al*. 2009 [58]	Case-control	33 MDD 16 Controls	*CD34* *CD133* *VEGFR2*	Diagnosis based on DSM-IV criteria BDI	Lower expression of *VEGFR2 *and *CD133 *in patients with MDD compared with controls. Expression of *VEGFR2 *and *CD133 *correlated negatively with BDI scores.
Anitha *et al*. 2008 [59]	Case-control	33 MDD 21 BPD 57 Controls	*PCNT2*	Diagnosis based on DSM-IV criteria BPRS HAM-D	Higher expression of *PCNT2 *in patients with MDD compared with controls. Higher expression of *PCNT2 *in patients with BPD in a remission state compared with controls.
Nakataki *et al*. 2011 [60]	Case-control	27 MDD 27 Controls	*EMP1*	Diagnosis based on DSM-IV criteria HAM-D	Lower expression of *EMP1 *in patients with MDD compared with controls. A trend increase in expression of *EMP1 *after 8 weeks of antidepressant treatment
Wakabayashi *et al*. 2008 [64]	Case-control	60 MDD 42 BPD 28 Controls	*NCAM-140* *L1* *VCAM-1* *ICAM-1* *E-cadherin*	Diagnosis based on DSM-IV criteria HDRS	Lower expression of *NCAM-140 *in patients with BPD in a current depressive state, but not in a remissive state, compared with controls and patients with MDD. Higher expression of *L1 *in patients with BPD in a current depressive state, but not in a remissive state, compared with controls and patients with MDD. No significant differences in expression of *NCAM-140 *or *L1 *in patients with MDD compared with controls. No significant differences in expression of *ICAM-1, VCAM-1 *or *E-cadherin *in patients with MDD or BPD in a current depressive state compared to controls.
Otsuki *et al*. 2010 [68]	Case-control	60 MDD 46 BPD 28 Controls	*REST* *CRH* *5-HT1A* *Adcy5* *CaMKIIa* *Epor* *IGF1R* *Tnfsf10* *Tnfsf11* *Tnfsf12-12*	Diagnosis based on DSM-IV criteria HDRS	Lower expression of *REST *in patients with MDD compared with controls. Higher expression of *CRH, Adcy5 *and *Tnfsf12-13 *in patients with MDD in a current depressive state compared with those in a remissive state. No significant difference in expression of REST or any other mRNAs in patients with BPD compared with controls.

BDI: Beck Depression Inventory; BPD: bipolar disorder; CARS-M: Clinician Administered Rating Scale for Mania; CDRS: Children Depression Rating Scale; DSM-IV: Diagnostic and Statistical Manual, Fourth Edition; HAM-D: Hamilton Rating Scale for Depression; HARS: Hamilton Anxiety Rating Scale; HDRS: Hamilton Depression Rating Scale; ICD-10: International Classification of Diseases - 10^th ^revision; MADRS: Montgomery-Asberg Depression Rating Scale; MDD: major depressive disorder; MINI: Mini International Neuropsychiatric Interview; SIGH-D-17: Structure Interview Guide for the 17-item Hamilton Depression Rating Scale; YMRS: Young Mania Rating Scale.

In a study of patients with MDD and BPD, Otsuki *et al*. did not find any significant differences in BDNF expression between patients and controls [49]. However, most of the patients were on antidepressant medication, so this may explain the lack of differences. Moreover, Otsuki and colleagues showed state-dependent differences in a number of other neurotrophic factors, including glial cell line-derived neurotrophic factor (GDNF), artemin (ARTN) and neurotrophin-3 (NT-3). These factors have previously been shown to be associated with stress response in animal models [50] as well as with depression and suicide in humans [51]. Specifically, they demonstrated that patients with MDD in a current depressive state have lower expression of GDNF, ARTN and NT-3 compared with those in remission as well as controls. However, they did not find any significant differences in the expression levels of these three factors in BPD patients in depressive or remissive states, suggesting that the changes in the expression of these genes are associated with MDD only, and may be state-dependent.

Another protein related to BDNF is p11, a member of the S-100 family known to be involved in the regulation of a number of cellular processes such as cell cycle progression and differentiation [52,53]. Interestingly, two studies have found p11 to be overexpressed in patients compared with healthy controls. Su *et al*. demonstrated that patients with MDD had a higher expression of p11 compared with controls [54], and Zhang *et al*. found the same results in patients with BPD [55]. However, in both of these studies the patients were medicated. Conversely, in our recent study we reported lower mRNA levels of p11 in drug-naïve patients with MDD compared with controls [23]. Furthermore, after 8 weeks of antidepressant treatment, p11 levels were significantly increased. We have also recently demonstrated that p11 mRNA levels are increased by antidepressant treatment *in vitro *in a human neuronal hippocampal model [43], thus showing also the unique ability of a gene expression approach to be used consistently across different experimental approaches.

As mentioned earlier, the expression of neurotrophic factors can be altered particularly in response to oxidative or reductive stress. One such neurotrophic factor is vascular endothelial growth factor (VEGF). Increased expression of VEGF has previously been shown in peripheral monocytes of patients with diabetes with coronary artery disease [56]. Given the high prevalence of depression in patients with coronary artery disease, VEGF mRNA levels have been proposed as a putative biological marker for MDD. Indeed, Iga and colleagues measured VEGF expression in the peripheral leukocytes of patients with MDD and showed that VEGF expression was higher in patients with MDD compared with healthy controls [57]. A similar study by Dome *et al. *[58] investigated the expression levels of VEGF receptor-2 (VEGFR2) in the peripheral blood of patients with MDD. They showed a lower expression of VEGFR2 in patients with MDD compared with healthy controls. Moreover, the expression of VEGFR2 negatively correlated with depression scores, thus supporting the role of VEGF signaling in MDD pathogenesis [58].

Two further molecules regulating neurogenesis have been found to be altered in depression: pericentrin 2 (PCNT2) and epithelial membrane protein 1 (EMP1). PCNT2 is a disrupted in schizophrenia 1-interacting protein that regulates cell proliferation, differentiation and migration, and outgrowth of neuronal axons and dendrites. In a study of patients with MDD and BPD, mRNA levels of PCNT2 were found to be significantly higher in drug-naïve patients with MDD compared with controls [59]. Interestingly, PCNT2 expression was also higher in patients with BPD in a remission state when compared with controls. EMP1 is involved in neurogenesis mechanisms as it interacts with transforming growth factor beta signaling. In drug-naïve patients with MDD, EMP1 levels were significantly lower when compared with controls and, after 8 weeks of antidepressant treatment, EMP1 mRNA levels showed a trend towards an increase [60].

Cell adhesion molecules such as neural cell adhesion molecule (NCAM) and L1 are also known to play important roles in synaptic plasticity, and have been indicated to have altered expression in the cerebrospinal fluid and brain of patients with a mood disorder [61-63]. Several studies conducted in peripheral blood mRNA confirm this. For example, Wakabayashi *et al. *[64] assessed the expression of NCAM-140 and L1 in the leukocytes of patients with MDD and BPD, as well as controls. They found a lower expression of NCAM-140 in patients with BPD in a current depressive, but not in a remissive, state compared with both controls and patients with MDD [64]. They also found a higher expression of L1, again in patients with BPD in a depressive state but not in those in remission compared with controls and patients with MDD. Interestingly, they did not find any significant differences in the expression of these molecules in patients with MDD when compared with controls. This suggests that the alterations in the expression of both NCAM-140 and L1 are specific to BPD and are also state dependent. In addition, no changes were found for intercellular adhesion molecule-1 (ICAM-1), vascular cell adhesion molecule-1 (VCAM-1) or E-cadherin expression, in patients with either MDD or BPD compared with controls.

Repressor element-1 silencing transcription factor (REST) is a modulator protein that is also known to be involved in synaptic plasticity [65]. It has been recently shown that REST is involved in the synthesis of cortisol [66] and in neurogenesis [67], both of which are of relevance to mood disorders. Otsuki and colleagues investigated the expression of REST and a variety of its target genes including corticotropin-releasing hormone (CRH), adenylate cyclise 5 (Adcy5) and TNF superfamily member 12-13 (TNFsf12-13) in patients with MDD and BPD [68]. They found a lower expression of REST in patients with MDD compared with controls. Furthermore, they investigated whether altered expression of these mRNAs were state or trait dependent, reporting a higher expression of CRH, Adcy5 and TNFsf12-13 in patients with MDD in a current depressive state compared with those in a remissive state. Interestingly, they found no significant differences in the expression of REST or any other mRNAs in patients with BPD when compared with controls.

## Transcriptomics studies

The use of high-throughput technologies like microarray platforms allows for the exploration of the expression levels of the entire genome and thus the identification of gene expression differences by using a hypothesis-free approach (See Table 4). Beech *et al*. used microarrays containing >48,000 transcript probes to investigate gene expression in the peripheral blood of patients with BPD compared with healthy controls [69]. They found a total of 1,180 differentially expressed genes, 559 of which were upregulated in patients with BPD and 621 that were downregulated. Using pathway analysis they were able to identify functional pathways that were significantly different between patients and controls, including pathways involved in gene transcription, immune response, apoptosis and cell survival. In particular, they found differences in the nuclear factor kappa-light-chain-enhancer of activated B cells (NF-κB) signaling pathway, which plays important roles in transcription regulation and immune response mechanisms. This is in line with a previous study showing increased DNA binding of NF-κB in peripheral blood mononuclear cells of patients with MDD in response to an acute stressor [70]. Another microarray study focusing on postpartum depression identified 73 differentially expressed genes in mothers with postpartum depression compared with control mothers [71]. Of interest, the authors observed a reduction in the expression of genes involved in immune modulation, transcriptional activation, cell cycle and proliferation, as well as DNA replication and repair processes. As previously mentioned, neuronal plasticity as well as cell survival are important processes involved in MDD and even in the effects of antidepressant drugs [72]. Indeed, one microarray study investigated gene expression changes in response to the serotonin-norepinephrine reuptake inhibitor venlafaxine in elderly patients with MDD [73]. The authors found 57 out of 8,000 sequences examined to have an altered expression after 4 weeks of antidepressant treatment. The genes found to be differentially expressed belong to the biological systems we have already discussed, including those involved in cell survival, ionic homeostasis, neural plasticity, signal transduction and metabolism. Lastly, a study in 2012 conducted microarray analysis in lymphocytes from patients with MDD and subsyndromal symptomatic depression (SSD) [74]. In patients with MDD, they found 149 differentially expressed genes, enriched in 53 pathways, in comparison with control participants. Pathway analyses identified significant differences for IL-2 and IL-6-mediated signaling as well as TNF receptor signaling pathways. In patients with SSD, they identified 1,456 genes and 47 pathways that were significantly different when compared with controls, with 20 genes overlapping with those found in patients with MDD. Pathways found to be differentially expressed in patients with SSD included cytokine-cytokine receptor interactions and G protein signaling. Only two pathways were found to be involved in both MDD and SSD: the mitogen-activated protein kinase signaling pathway and the Wnt signaling pathway, both of which have been previously implicated in mood disorders [75,76].

**Table 4:** Studies using transcriptomics

Citation	Type of study	Sample	GENE Technique	Clinical assessment	Main findings
Beech *et al*. 2010 [69]	Case-control	20 BPD 15 Controls	Microarray	Diagnosis based on DSM-IV criteria SCID HDRS MADRS HARS YMRS	Altered expression of 1180 genes in patients with BPD compared with controls. Higher expression of 559 genes and lower expression of 621 genes. Top three functional pathways affected were the mitochondrial electron transport chain, notch signaling and NF-κβ signaling pathways.
Segman *et al*. 2010 [71]	Case-control	10 mothers with postpartum depression 10 control mothers	Microarray	Diagnosis based on DSM-IV criteria SCID EPDS	Altered expression of 73 genes in mothers with postpartum depression compared with controls mothers. Lower expression of 71 genes in mothers with postpartum depression including genes related to transcriptional activation, cell cycle and proliferation, nucleotide binding, and DNA replication and repair.
Kalman *et al*. 2005 [73]	Repeated measures	6 elderly MDD	Microarray	Diagnosis based on ICD-10 criteria BDI HAM-D GDS MMSE	Altered expression of 57 genes after 4 weeks of venlafaxine treatment when compared to baseline expression levels. Lower expression of 26 and higher expression of 31 including genes involved in cell survival, ionic homeostasis, neural plasticity, signal transduction and metabolism
Yi *et al*. 2012 [74]	Case-control	8 MDD 8 SSD 8 Controls	Microarray	Diagnosis based on DSM-IV criteria HDRS-17	Altered expression of 149 genes in patients with MDD and 1,456 genes in patients with SDD compared with controls. Functional pathways affected included IL-2- and IL6-mediated signaling and TNF receptor signaling in patients with MDD and cytokine-cytokine receptor interactions and G protein signaling pathway in patients with SDD.

BDI: Beck Depression Inventory; BPD: bipolar disorder; BPRS: Brief Psychiatric Rating Scale; CIDI: Composite International Diagnostic Interview; DSM-IV: Diagnostic and Statistical Manual, Fourth Edition; EPDS: Edinburgh Postnatal Depression Scale; GDS: Geriatric Depression Scale; HAM-D: Hamilton Rating Scale for Depression; HDRS: Hamilton Depression Rating Scale; ICD-10: International Classification of Diseases - 10^th ^revision; MADRS: Montgomery-Asberg Depression Rating Scale; MDD: major depressive disorder; MMSE: Mini-Mental State Examination; SCAN: Schedules for Clinical Assessment in Neuropsychiatry; SCID-I: Structured Clinical Interview for DSM-IV Axis I Disorder; SSD: subsyndromal symptomatic depression; YMRS: Young Mania Rating Scale.

Although strictly speaking not a transcriptomics study in patients with depression, it is worth mentioning our recent study in the human hippocampal cells model [77]. In this study, we mimicked depression 'in a dish' by incubating cells with stress-level concentrations of the main human glucocorticoid hormone, cortisol. Transcriptomics analyses have identified inhibition of the 'Hedgehog pathway' as a candidate mechanism by which depression can reduce neurogenesis. It is of interest that, in the same study, we also found that Hedgehog-signaling is inhibited in the hippocampus of adult prenatally stressed rats with high glucocorticoid levels, again confirming the ability of gene expression approach to identify findings that are replicated consistently across different experimental models.

## Conclusions

We have presented data on peripheral mRNA gene expression in patients with depression across MDD and BPD, obtained from whole blood, isolated mononuclear cells and isolated monocytes. All studies identified a pattern of altered expression in several genes belonging to three biological systems of interest: inflammation, GR functionality and neuroplasticity. Of note is the frequent pattern of state-related gene expression changes that are normalized either by remission or by antidepressant treatment. The association between gene expression and treatment response identifies this biomarker approach as particularly relevant from a clinical point of view. However, the temporal relationship of these gene expression changes with other factors, such as exposure to stress, is still unclear. This is relevant especially considering the frequent occurrence of stressors in these clinical groups. For example, a study on socioeconomic circumstances used transcriptome gene expression measurements followed by bioinformatics analysis of genes whose expression is regulated by specific transcription factors, including the GR and NF-κB. The authors described an upregulation of target genes for NF-κB and a downregulation of target genes for GR, consistent with a pattern of glucocorticoid resistance and increased inflammation, that is, a pattern similar to that described in depression [78]. We also do not know whether some of these changes in gene expression represent the marker of a genetic predisposition to encounter psychopathology; for example, we have previously shown that genetic variants in CNS and immune genes increase the association between depression and inflammation [79].

It should also be noted that the many pathways involved in the onset of depressive symptoms are of course interrelated and dynamic in nature. Because of this complexity, it has been proposed that a systems biology approach, combining information from gene expression analysis, protein data and well-validated animal models, is necessary to untangle the exact relevant pathways as well as novel molecular mechanisms [80]. Despite these unanswered questions, peripheral blood gene expression is a strong and clinically relevant system to identify biomarkers related to pathology and treatment response, and also to discover unknown mechanisms underlying the development of mood disorders. The identification of both could help in the personalization of therapy and in the future development of novel treatments.

## Abbreviations

Adcy5: adenylate cyclise 5; ApoE: apolipoprotein E; ARTN: artemin; BAG1: BAG family molecular chaperone regulator 1; BDNF: brain-derived neurotrophic factor; BPD: bipolar disorder; CNS: central nervous system; COX-2: cyclooxygenase-2; CRH: corticotropin-releasing hormone; DAP12: DNAX-activation protein of 12 kDa; EMP1: epithelial membrane protein 1; FKBP: FK506 binding protein; GDNF: glial cell line-derived neurotrophic factor; Glo1: glyoxalase-1; GR: glucocorticoid receptor; HPA: hypothalamic-pituitary-adrenal; ICAM: intercellular adhesion molecule-1; IFN-γ: interferon gamma; IL: interleukin; MDD: major depressive disorder; MIF: macrophage inhibiting factor; MPO: myeloperoxidase; NCAM: neural cell adhesion molecule; NCOA1: nuclear receptor coactivator 1; NF-κB: nuclear factor kappa-light-chain-enhancer of activated B cells; NT-3: neurotrophin-3; PCNT2: pericentrin 2; PLA2G2A: phospholipase A2; PPID: peptidylprolyl isomerase D; PU.1: purine-rich Box-1; REST: repressor element-1 silencing transcription factor; SSD: subsyndromal symptomatic depression; TNF-α: tumor necrosis factor alpha; TNFsf12-13: tumor necrosis factor superfamily member 12-13; TREM-1: triggering receptor expressed on myeloid cells 1; VCAM-1: vascular cell adhesion molecule-1; VEGF: vascular endothelial growth factor; VEGFR2: vascular endothelial growth factor receptor-2.

## Competing interests

CMP in the last 3 years has received fees as a speaker or as a member of advisory boards, as well as research funding, from pharmaceutical companies that commercialize or are developing antidepressants, such as Lilly, Servier and Janssen. PAZ received fees as a speaker from Servier. All other authors declare no conflict of interest.

## Authors' contributions

NH and CMP conceptualized this work, conducted the literature search and drafted the initial manuscript. AC and PAZ significantly contributed to the subsequent revisions, and gave approval to submit the final version. All authors read and approved the final manuscript.

## Pre-publication history

The pre-publication history for this paper can be accessed here:

http://www.biomedcentral.com/1741-7015/11/28/prepub
